# Association between iron deposition in splenic,hepatic and myocardial tissues assessed by T2* relaxometry technique

**DOI:** 10.22088/cjim.12.4.600

**Published:** 2021

**Authors:** Ali Mohammadzadeh, Saeed Alizadeh, Layla Shojaie, Maryam Mohammadzadeh

**Affiliations:** 1 Iran University of Medical Sciences, Rajaie Hospital, Tehran, Iran; 2 Department of Radiology, Iran University of Medical Sciences, Rajaie Hospital, Tehran, Iran; 3 Tehran University of Medical Sciences, Tehran, Iran

**Keywords:** Magnetic Resonance Imaging, T2* Mapping, Spleen, Beta-Thalassemia, Iron Concentration

## Abstract

**Background:**

Decreasing signal intensity of the spleen assessed by T2* MRI is a frequent finding in patients with beta-thalassemia due to iron deposition within the reticuloendothelial cells in this organ. This parameter can also be applied to determine the candidates for blood cell transfusion. However, the association between splenic siderosis and iron overload in other vital organs such as heart and liver remains unclear. The present study aimed to assess the correlation between iron deposition in splenic, hepatic and myocardial tissues by T2* relaxometry technique.

**Methods:**

This cross-sectional study included 39 consecutive patients with a definitive diagnosis of beta-thalassemia major who underwent spleen, liver and heart MRI examinations for iron deposition and cardiac function.

**Results:**

No significant correlation was found between the heart and splenic T2* relaxation time (R=0.206, P=0.357). We revealed a strong correlation between the splenic T2* relaxation time and hepatic calculated T2*s (R=0.515, P=0.014). The liver T2* values can be predicted from the splenic T2*s by a new linear equation. According to the ROC curve analysis, the splenic T2* could significantly, but moderately predict moderate to severe from mild liver iron excess (AUC=0.667).

**Conclusion:**

Our study demonstrated a significant linear correlation between the splenic and hepatic T2* relaxation time, probably indicative of the same iron deposition mechanism, and made us available to write a linear model that would predict the deposited iron density in the spleen with the use of the magnetic resonance T2* values.

In beta-thalassemia patients, varying mechanisms, transfusion and non-transfusion-dependent, are responsible for iron loading. This phenomenon can be first developed by increasing iron absorption from the intestine signalled by ineffective erythropoiesis that might be secondary to transfusion (1). Suppressing serum hepcidin also leads to increase iron absorption and, consequently releasing iron from the reticuloendothelial system leading iron overload in vital organs such as liver (2, 3). Overall, in both transfusion and non-transfusion-dependent thalassemia, iron overloading can result in adverse clinical consequences, including bone disorders, pulmonary hypertension, endocrinopathy, metabolic disorders, cerebrovascular damages, liver fibrosis or cirrhosis, and even malignancies such as hepatocellular carcinoma (4-6). Dramatically, such damages might be impacted by some medications such as iron chelators that are eventually used to manage iron overload (7).

More than all, heart and liver are significantly affected by iron overloading. The occurrence of cardiac iron overloading commonly leads to cardiac siderosis, a severe source of cardiac arrhythmia, heart failure, and even death, especially in patients with transfusion-dependent thalassemia (8). In this regard, reduction of cardiac-related mortality following proper management of cardiac iron overload can be expected (9). 

More interestingly, in non-transfusion-dependent thalassemia, the liver is involved rather than the myocardium leading to liver fibrosis and chronic, increase the risk for viral hepatitis cirrhosis and even hepatic failure (10).

Along with clinical evaluation, some recent imaging techniques such as magnetic resonance imaging using R2 or T2* techniques have been introduced as a good alternative for invasive management via biopsy for the assessment of iron overload (11).

 Although histological assessment has been known as a highly sensitive and specific for assessing iron overload in vital organs, the diagnostic performance of R2 or T2* MRI in this goal has received strong attention (12). MRI is able to quantify both liver and cardiac iron concentration, and in this regard, this technique has also been considered as an accurate and safe technique for monitoring iron overloading in such vital organs (13).

As a new suggestion, because the spleen and lymph nodes are reticuloendothelial cells-rich organs, the effect of these organs via iron overload is also expected (14); however, few studies assessed the iron overload in these organs through MRI among the thalassemia patients. Some evidence is available in the association between serum ferritin level and loading of iron in spleen assessed by the Signal Intensity Ratio (SIR) method (15). However, there is some evidence against this claim. In this regard, some authors could not find any association between liver and spleen siderosis in the background of thalassemia, probably due to the differences in iron kinetics in both organs (16). In total, the assessment of the association of iron overloading in cardiac and hepatic tissues and spleen by SIR can lead to a better understanding of the pathophysiology of the iron overloading and its sequels and, therefore a better treatment protocol in thalassemia. The present study aimed to assess the correlation between iron deposition in splenic, hepatic and myocardial tissues by T2* relaxometry technique. Our finding can also help to present a linear relationship between the splenic iron density and its T2* values. 

## Methods

**Study Population: **This cross-sectional study included 39 consecutive patients with a definitive diagnosis of beta-thalassemia major who underwent spleen, liver, and heart MRI examinations for iron deposition and cardiac function. All national and international ethical research rules were supervised by the institution's ethics research committee, and the researchers followed the rules completely. 

**MRI Examination: **All the MRI examinations were performed using a 1.5 Tesla MR system (Avanto, Siemens, Erlangen, Germany). The protocol was identical for all patients. A 16-channel phased-array body Radiofrequency (RF) coil was employed for the signal reception. All the sequences were ECG-gated either retrospectively or prospectively. For cine imaging, several 2D, Breath-held, True-Fast Imaging with Steady State Precession MRI (True-FISP) were performed retrospectively in the 2-chamber, 4-chamber, and a whole-heart coverage short-axis views in all the patients assess- ing the left ventricular function qualitatively and quantitatively tFor T2* mapping, the cardiac T2* relaxometry data were acquired by a breath-held, Bright-Blood, Gradient-Recalled-Echo pulse sequence which could obtain 8 signals with different echo-times (TE) in each repetition-time (TR). The patients' hearts were imaged in the three cardiac basal, mid-ventricular, and apical short-axis, and three spleen and hepatic transverse planes by the given parameters (table 1). Region of interests (ROIs) are the parts mostly in the parenchyma and far from the vessels so we can avoid blood and have an accurate measurement. The acquired left-ventricular functional images were analyzed both qualitatively and quantitatively by two experts in cardiothoracic radiology using CMR42 (Circle Cardiovascular Imaging, Calgary, Canada) cardiac MRI analysis software. The T2* relaxation times were quantified by an experienced clinical MRI physicist in the whole inter-ventricular septal wall by the same software too. Finally, all the mean T2* values, and the associations between the values were analyzed statistically.

**Statistical analysis: **The results were presented as mean ± standard deviation (SD) for quantitative variables and were summarized by absolute frequencies and percentages for categorical variables. Normality of data was analyzed using the Kolmogorov-Smirnoff test. The association between the quantitative variables was examined by the Pearson’s correlation test or Spearman’s non-parametric correlation test. The receiver operating characteristic (ROC) curve analysis was employed to assess the value of splenic T2* in predicting the severity of iron excess in the heart and liver. The Statistical Package for the Social Sciences (SPSS), (IBM Corp. Released 2013, IBM SPSS Statistics for Windows, Version 22.0. Armonk, NY: IBM Corp) was used for analysis. P-values of 0.05 or less were considered statistically significant. All the data used in the analysis were labeled, hence the statisticians were blind to the groups.

## Results

**Fig (1):**
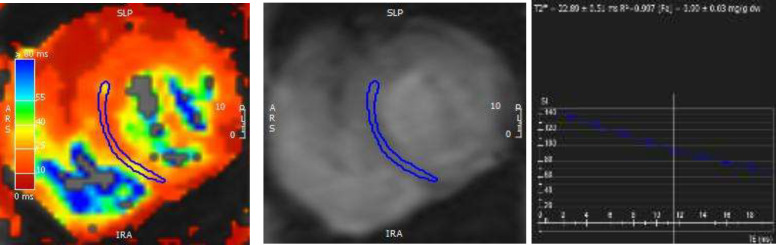
The patient with major beta thalassemia myocardial signal decay curve shows no significant iron deposition in heart (T2* = 22.89 +/- 0.997)

**Fig (2):**
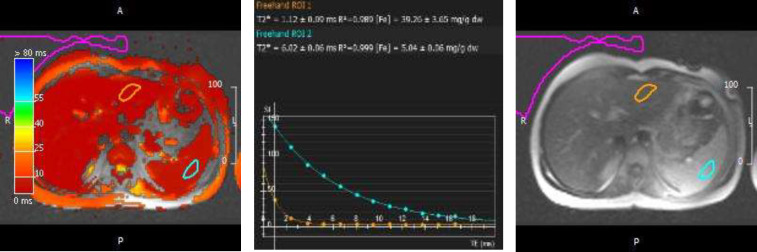
The same patient in figure 1 As it can be noticed from the hepatic and splenic signal decay curves, There is a significant correlation between the mentioned tissues iron overload (blue: splenic T2* decay curve with the value of 6.02 +/- 0.06, orange: hepatic T2* decay curve with the value of 1.12 +/- 0.09). Despite of a strong hepato-splenic iron deposition correlation, there is no significant relationship with cardiac iron overload (in Fig 1)

Overall, 98 region of interests (ROIs) were drawn for all patients who participated in our study. 37 ROIs were drawn in liver (mean: 4.1868±5.1317 ms), out of which 23 ROIs were classified as the severe iron overload, 10 ROIs with moderate iron overload, and 4 with mild iron status. Also, 38 ROIs were drawn in the myocardial inter-ventricular septum (mean: 23.3776±11.3660 ms), out of which, 7 ROIs were diagnosed with severe iron deposition and 31 with mild status. No moderate myocardial iron deposition was noticed among the patients. From the total 98 regions of interest, 21 ROIs were drawn in the middle of the splenic tissue (mean: 16.0136±17.0022 ms). Due to the non-parametric nature of all quantitative parameters assessed, the bivariate two-tailed Spearman’s correlation test was performed between the splenic and myocardial iron deposition statuses (figure 1) and indicated no significant correlation between the heart and splenic T2* relaxation times (R=0.206, P=0.357). The splenic and hepatic T2* relaxation value correlation was also tested by the same method to find any iron deposition correlation between the organs. The Spearman’s statistical analysis revealed a strong correlation between the splenic T2* relaxation times and hepatic-calculated T2*s (R=0.515, P= 0.014) (figure 2). The results revealed strong evidence of the splenic and hepatic iron concentrations correlation. The liver T2* values can be predicted from the splenic T2*s by the below linear equation:$T_{2(\mathrm{Liver})}^{*}=0.022T_{2(\mathrm{Splenic})}^{*}+1.144$

**Figure 3:**
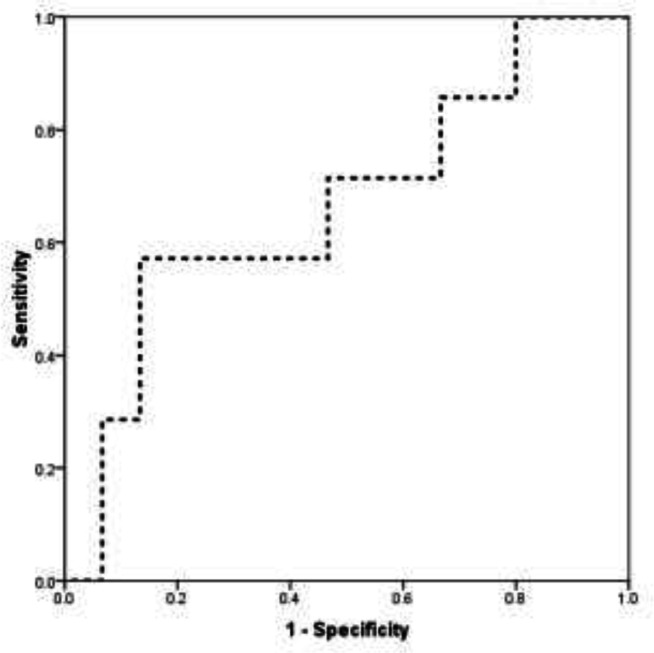
The area under the ROC curve to determine the value of splenic T2* for predicting moderate to severe from mild liver Iron excess

According to the ROC curve analysis (figure 3), the splenic T2* could significantly, but moderately predict moderate to severe from mild liver iron excess (AUC = 0.667, 95%CI: 0.412 – 0.922). The best cutoff value of splenic T2* for predicting moderate to severe hepatic iron excess was 6.25 yielding a sensitivity of 71.4% and a specificity of 53.3%. 

**Table 1:** Sequence description and parameters used in this study

**Sequence**	**True-FISP**	GRE- 8 Echoes*
TR/TE (ms)	70.5/1.19	200/(2.59,4.82,7.05,9.28,11.51,13.74,15.97,18.20)
Flip Angle	65	20
FOV (mm)	320*260	300*400
Aq. Matrix Size	140*192	86*192
Pixel Size (mm)	1.9*1.7	3.5*2.1
BW (Hz/Px)	930	810
Averages	1	1
ECG-Gating	retrospective	Prospective-Systole
Application	sine	T2* Mapping

*GRE-8 Echoes: Gradient Recalled Echo with 8 TEs.

## Discussion

Decreasing signal intensity of the spleen assessed by T2* MRI is a common finding in patients with beta-thalassemia frequently due to iron deposition within the reticuloendothelial cells in this organ (17, 18). This parameter can also be applied to determine candidates for blood cell transfusion. However, the association between splenic siderosis and iron overload in other vital organs such as heart and liver remain unclear. In this regard, even in some patients with normal splenic signal intensity, hypointense liver was revealed (19, 20). It seems that iron sourced from red blood cells can accumulate in the reticuloendothelial cells of the liver, spleen, and myocardium due to high oxygen demands of such organs [21]; however, some disagreements between loads of iron in these three organs should be scientifically explained. The present study could well show a strong and direct association between iron overload assessed by T2* values in liver and spleen revealing high value of assessing splenic iron overload to predict liver iron over-excess, however, such association was not found between splenic iron overload and iron deposition in myocardium. A few studies assessed such associations. In a study by Papakonstantinou et al. in 2009 (16), and similar to our study, hepatic R2 values correlated with splenic but not with myocardial R2 values. In a study by Brewer et al. in 2009 (22), the strong association was found between splenic and liver iron level only in those sickle cell disease patients but not in thalassemia major group. In another study by İdilman et al. in 2016 (23), the liver R2* was only associated with pancreas R2* and renal cortex R2*, but not with spleen R2*. Despite this fact that high iron deposit is found in the reticuloendothelial cells in spleen, some earlier studies could show that iron deposited in the reticuloendothelial cells of the spleen is rapidly removed and redistributed throughout the body leading ultimately low splenic content of iron in thalassemia (24). Thus, iron deposited in the spleen may be an indicative for hepatic iron excess, but not in all disease subgroups. In other words, the baseline characteristics of the patients may affect such association. 

MRI plays a pivotal role in planning the beta-thalassemia major treatment strategy. The hepatic biopsy is the gold standard method to measure the iron deposition density; however, as an invasive method, it has limitations for repeated assessment. Hence, MR relaxometry could be a suitable tool for patients' annual assessment. There were numerous studies evaluating the role of MRI and its accuracy in in-vivo iron measurements by the use of T1, T2, and T2* relaxometry methods. By recent investigations, it has been proven that the T2* relaxation time was the most robust phenomenon by the iron deposition density. In the recent years, many studies have been done on the hepatic and myocardial iron deposition density and kinetics by the use of T2* relaxometry, but few were done on spleen and the endocrine system. In this study, we assessed the linear/curvilinear relationship between the splenic and hepatic/myocardial T2* relaxation times, to find the similarities of iron deposition mechanisms and acquire a robust relationship between the splenic iron content and its quantitative T2* values based on the previous studies. Our study demonstrated a significant linear correlation between the splenic and hepatic T2* relaxation times, probably indicative of the same iron deposition mechanism, and made us available to write a linear model that would predict the deposited iron density in spleen by the use of the magnetic resonance T2* values.

**Ethical considerations: **The protocol of the study was approved by the Ehics Committee of Iran University of Medical Sciences with the number of IR.IUMS.REC.1396.9211282015. Design and objectives of the study were explained to the parents and written informed consent was obtained from those who were willing to participate in the study and they were ensured that their information will be kept confidential and analyzed anonymously. All the ethical considerations of Helsinki declaration were met throughout the study.
